# The function of Mgs1/WRNIP1 in genome maintenance

**DOI:** 10.3389/fmolb.2026.1923317

**Published:** 2026-08-27

**Authors:** Sara Afzali Jaktajdinani, Lili Hegedűs, Nargis Karatayeva, Roberta Fajka-Boja, Peter Burkovics

**Affiliations:** 1 Laboratory of Replication and Genome Stability, Institute of Genetics, HUN-REN Biological Research Centre, Szeged, Hungary; 2 Doctoral School of Biology, University of Szeged, Szeged, Hungary; 3 Laboratory of DNA Damage and Nuclear Dynamics, Institute of Genetics, HUN-REN Biological Research Centre, Szeged, Hungary; 4 Department of Immunology, Albert Szent-Györgyi Medical School, Faculty of Science and Informatics, University of Szeged, Szeged, Hungary

**Keywords:** DNA repair, DNA replication, Mgs1, stalled replication fork, WRNIP1

## Abstract

Preservation of genomic integrity during replication is challenging, because replication forks are often stalled by several forms of DNA damage or stable secondary DNA structures. Prolonged stalling of the replication fork can lead to incomplete replication, which may induce double strand breaks, genome rearrangements and cell death. Therefore, several DNA repair mechanisms evolved to rescue stalled replication forks, which can elaborate in error-free or error-prone manners. The pathway selection and the fine tuning of the collaboration between different DNA repair proteins involved in the rescue of the stalled replication fork are very important. Based on our recent knowledge, yeast Mgs1 and its human homologue WRNIP1 proteins can be excellent candidates for this fine-tuning regulator function. In this review we summarize our current knowledge about them and try to point out the most important future steps to prove this hypothesis.

## 1 Introduction

Maintenance of genomic integrity is essential for the survival and proper functioning of all living organisms. Accurate duplication of the genetic information during DNA replication represents a major challenge since replication machinery continuously encounters obstacles that can impede the replication process (Chatterjee and Walker, 2017). Although the selective pressure acting on genome maintenance differs between unicellular organisms, such as *Saccharomyces cerevisiae,* and multicellular organisms, such as humans, efficient mechanisms that preserve replication fork integrity are indispensable in both systems. In a single-cell organism, the completion of replication, cell division, as well as preventing cell death, have to be ensured at all costs, but in a multicellular organism, the selection pressure on a single cell division is lower.

To cope with replication-associated DNA damage and replication stress, cells have evolved several different DNA repair and damage tolerance mechanisms, including non-homologous end joining (NHEJ), homologous recombination (HR), DNA damage tolerance (DDT) pathway, and the Fanconi anaemia (FA) pathway (Moldovan and D’Andrea, 2009; Jasin and Rothstein, 2013; Yeeles et al., 2013; Scully et al., 2019). These pathways coordinate the stabilization, processing and restart of stalled or collapsed replication forks. Dysregulation or failure of these pathways results in genome instability, hereditary genetic disorders and cancer development in humans (Torgovnick and Schumacher, 2015).

While the core components of replication stress response and DNA repair pathways have been well characterized, the role of accessory regulatory factors, involved in the fine-tuning of fork rescue mechanisms remain incompletely understood. Among these factors are Werner helicase interacting protein 1 (WRNIP1) and its yeast homologue Mgs1, members of the evolutionary conserved AAA^+^ ATPase family (Hishida et al., 2001; Yi et al., 2001; Paeschke and Burkovics, 2021). Increasing evidence indicates that these proteins play important roles in replication fork metabolism, genome stability maintenance, and the coordination of the DNA damage tolerance pathways (DDT). In this review, we summarize the current understanding of the functions of WRNIP1 and Mgs1 and discuss the mechanistic similarities between the yeast and human proteins in the maintenance of genomic integrity. We discuss their roles in replication stress responses, fork protection, and the regulation of DNA repair and DDT.

## 2 The evolutionary conservation of the Mgs1/WRNIP1 protein

Yeast Mgs1 ATPase is implicated in replication-associated genome maintenance pathways. Sequence analyses revealed that the central ATPase region of yeast Mgs1 shares similarity with bacterial RuvB-like ATPases and replication factor C (RFC)-related ATP-binding proteins, including the conserved Walker A/B and sensor motifs characteristic of the AAA+ (ATPases Associated with diverse cellular Activities) ATPase superfamily (Hishida et al., 2001). Biochemical studies later demonstrated that Mgs1 possesses DNA-dependent ATPase activity and promotes single-stranded DNA annealing (Hishida et al., 2001), supporting its proposed role in replication fork metabolism and DDT. Genetic studies in *Saccharomyces cerevisiae* further demonstrated that loss of Mgs1 results in increased genome instability and increased mitotic recombination (Hishida et al., 2001).

RecQ family DNA helicases play essential roles in homologous recombination and in replication-associated DNA repair. In yeast, Sgs1 is the functional representative of this family DNA helicases. In humans, five RecQ family helicases have been identified, one of which is the Werner syndrome helicase (WRN). Mutations in the WRN gene cause Werner syndrome, a rare premature aging disorder, characterized by genomic instability (Hanada and Hickson, 2007; Gupta and Schmidt, 2020). Notably, simultaneous deletion of MGS1 and SGS1 results in severe synthetic growth and genome maintenance defects, indicating that these proteins perform partially overlapping functions in the cellular responses to replication stress (Hishida et al., 2001; Branzei et al., 2002b).

**FIGURE 1:**
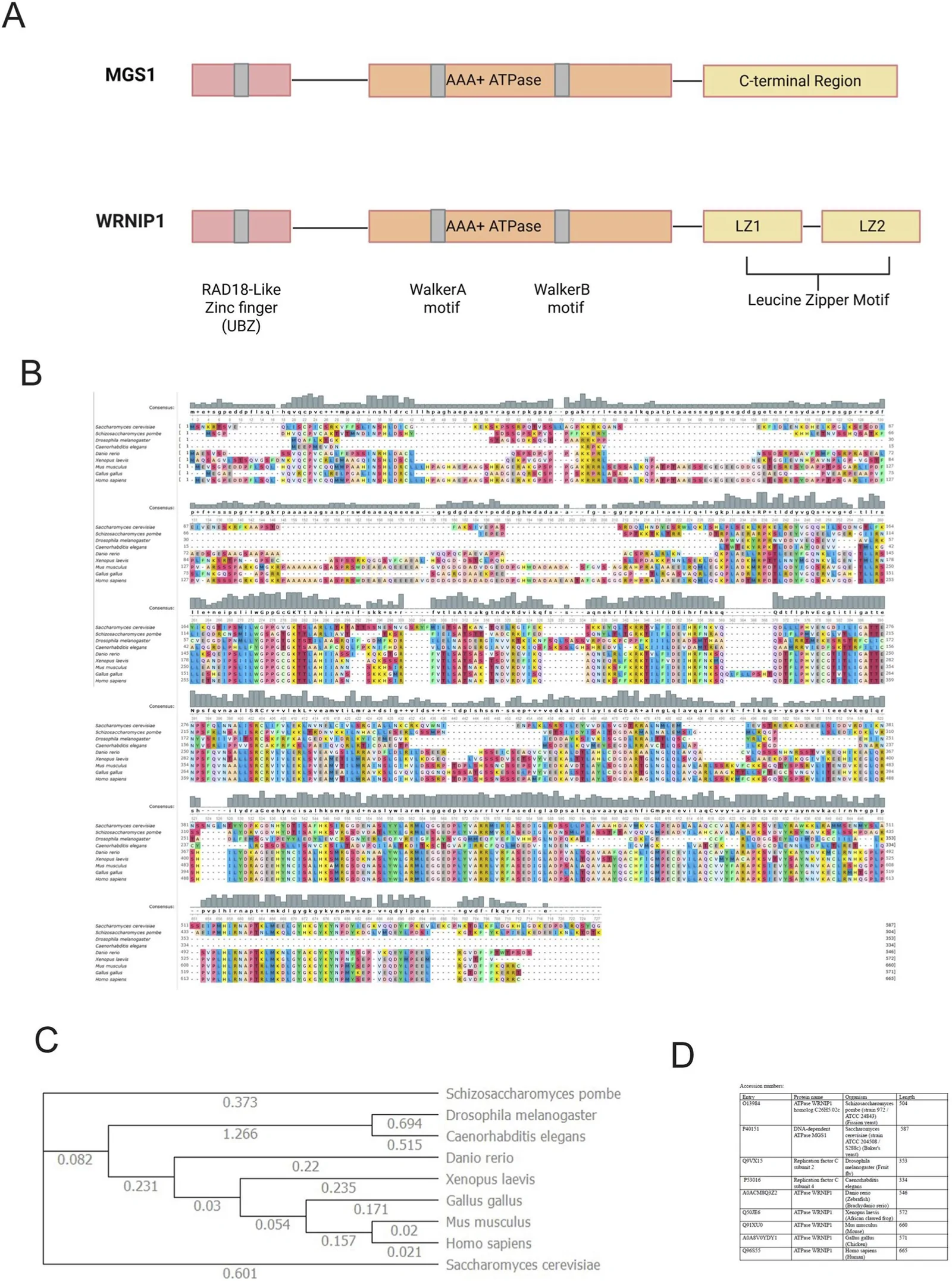
Mgs1/WRNIP1 proteins are evolutionary conserved. **(A)** Domain structure of Mgs1 and WRNIP1 proteins. **(B)** Sequence alignments of Mgs1/WRNIP1 proteins from different species. To analyse the evolutionary conservation of the Mgs1/WRNIP1 protein, full-length amino acid sequences for nine representative eukaryotic species were retrieved from the UniProt database. Multiple sequence alignment (MSA) was performed using the MUSCLE (Multiple Sequence Comparison by Log-Expectation) algorithm implemented in the UGENE bioinformatics platform. The resulting alignment matrix was visualized using a standard property-based scheme to highlight the identity and chemical similarity of amino acids. **(C)** To evaluate the evolutionary relationships of the Mgs1/WRNIP1 protein, a phylogenetic tree was constructed using the full-length amino acid sequence alignment of nine representative eukaryotic species. The tree topology and branch lengths were calculated using the PHYLIP Neighbor-Joining (NJ) algorithm integrated within the UGENE bioinformatics toolkit. The resulting tree was visualized in UGENE. **(D)** List of the accession numbers, used for the analyses.

The human homologue of Mgs1, initially identified as WHIP (Werner helicase-interacting protein) and later renamed WRNIP1, was discovered through its interaction with the Werner syndrome helicase WRN in yeast two-hybrid assays (Yi et al., 2001). Subsequent studies established WRNIP1 as a multifunctional replication stress response factor involved in replication fork stabilization (Leuzzi et al., 2016; Porebski et al., 2019; Shi et al., 2023), DNA damage tolerance (Yoshimura et al., 2006; 2014; Hayashi et al., 2008), and checkpoint-associated pathways in vertebrate cells (Kanu et al., 2016). Mgs1 and WRNIP1 display a highly conserved domain organisation across eukaryotes (Figure 1). They contain an N-terminal ubiquitin-binding zinc finger (UBZ) domain, a central AAA + ATPase domain, and a C-terminal leucin zipper domain (Yoshimura et al., 2017; 2022) (Figure 1A). The PCNA (Hishida et al., 2006) binding site (PIP motif) and the nuclear localization signal were also identified in the Mgs1/WRNIP1 sequence (Jordan et al., 2023). The evolutionary conservation of this structure suggests an important and conserved role of these proteins in genome maintenance (Figure 1).

The alignment revealed that the core AAA + ATPase domain, located in the central-to-C-terminal region, is highly conserved across all nine species analysed (Figure 1B). However, the UBZ domain is missing from *Caenorhabditis elegans* and *Drosophila melanogaster* sequences (Figure 1B), indicating the possibility that the Mgs1/WRNIP1 is not functional in these two species. This result is not surprising, because Rad18 is also missing from *D. melanogaster* (Sekelsky, 2017) and not described in *C. elegans*, too. These findings also indicate that one of the main functions of Mgs1/WRNIP1 is tightly associated with the function of RAD6/RAD18 ubiquitin ligase complex as described in yeast (Hishida et al., 2002; 2006).

The phylogenetic analysis reveals that Mgs1/WRNIP1 are highly conserved across the eukaryotic taxa (Figure 1C). As expected, the fungal homologues of *Saccharomyces cerevisiae* and *Schizosaccharomyces pombe* form a distinct clade that is separate from the metazoans. Within the metazoans, the overall tree topology mostly follows the established species relationships, with vertebrate homologues clustering according to the evolutionary history (from fish through amphibian and avian intermediates to mammals). Human and mouse WRNIP1 proteins display very short branch lengths, 0.021 and 0.020, respectively, which indicates a highly conserved sequence relationship between mammalian orthologues. In contrast, *C. elegans* exhibits a significantly greater sequence divergence with the branch length of 1.266. Even though this might reflect lineage-specific evolutionary divergence, it does not necessarily imply functional divergence, and experimental evidence on the role of *C. elegans* Mgs1/WRNIP1 is limited.

## 3 UBZ domain of Mgs1 and WRNIP1

Mgs1 and WRNIP1 contain an N-terminal ubiquitin-binding zinc finger (UBZ) domain, located approximately within amino acids 20–80. It belongs to the CCHC-type UBZ family, which was originally identified in Y-family translesion synthesis polymerases (Bienko et al., 2005). Structurally, UBZ domains form zinc-coordinating β-β-α fold motifs, that are predominantly found in proteins involved in DNA repair, replication stress responses and transcription-associated regulatory pathways (Suzuki et al., 2016). Zinc ion coordination through conserved cysteine and/or histidine residues, enables the recognition and binding of ubiquitin (Ub) moieties (Hofmann, 2009).

The UBZ domain of Mgs1/WRNIP1 has been implicated in the recognition of ubiquitinated proliferating cell nuclear antigen (PCNA) (Hishida et al., 2006; Bish and Myers, 2007; Saugar et al., 2012; Yoshimura et al., 2022), a central coordinator of DDT pathways. Biochemical studies demonstrated that Mgs1 preferentially interacts with polyubiquitinated PCNA, whereas binding to monoubiquitinated PCNA or free ubiquitin is considerably weaker (Saugar et al., 2012). These findings suggest that Mgs1, and potentially WRNIP1, function during template-switching-associated DNA damage tolerance rather than translesion synthesis pathways.

In human cells, WRNIP1 itself undergoes polyubiquitination and multi-monoubiquitination in a UBZ-domain-dependent manner (Bish et al., 2008). This observation raises the possibility that WRNIP1 may participate in intra- or intermolecular ubiquitin-dependent regulatory interactions through recognition of its own ubiquitin chains. Such a mechanism could potentially affect WRNIP1 localization, protein-protein interactions, or replication stress response functions (Bish and Myers, 2007; Crosetto et al., 2008; Nomura et al., 2012; Socha et al., 2020; Yoshimura et al., 2022; Valenzisi et al., 2024). In contrast, ubiquitination of yeast Mgs1 has not yet been conclusively demonstrated.

## 4 The ATPase domain of Mgs1 and WRNIP1

The mechanistic role of the ATPase activity of human WRNIP1 remains less extensively characterized. Nevertheless, accumulating evidence suggests that the ATPase domain contributes to replication fork restart, replication stress responses, and the regulation of interactions with specific replication-associated protein partners (Hishida et al., 2001; Branzei et al., 2002b; Kim, 2005; Tsurimoto et al., 2005; Kawabe et al., 2006; Vijeh Motlagh et al., 2006; Yoshimura et al., 2009; Kanamori et al., 2011; Leuzzi et al., 2016; Jiménez-Martín et al., 2020; Yoon et al., 2025).

In the central region of both Mgs1 and WRNIP1, the highly conserved AAA + ATPase domain contains the canonical Walker A (P-loop) and Walker B motifs, which mediate ATP binding and ATP hydrolysis, respectively. Within the Walker A motif, the conserved lysine residue is essential for ATP binding, whereas a conserved glutamate residue within the Walker B is required for ATP hydrolysis (Iyer et al., 2004).

Functional studies in yeast demonstrated that mutations affecting either the Walker A or Walker B motifs impair multiple Mgs1 functions, including DDT and resistance to replication-associated stress (Hishida et al., 2001; Branzei et al., 2002b; Kim, 2005; Vijeh Motlagh et al., 2006; Jiménez-Martín et al., 2020). These findings indicate that ATP binding and hydrolysis are essential for the biological activity of Mgs1 during replication fork maintenance and genome stability preservation.

## 5 The C terminus and leucine zipper domain of Mgs1 and WRNIP1

In the current models, the C-terminal region of both Mgs1 and its mammalian homolog WRNIP1 primarily contributes to regulatory and protein–protein interaction functions. However, compared to the N-terminal UBZ domain and the central AAA + ATPase region, the C terminal part of Mgs1 remains relatively poorly characterized.

In WRNIP1, the C-terminal region (approximately amino acids 450-665) contains leucine zipper motifs composed of periodically repeated leucine residues that are predicted to facilitate protein oligomerization and the formation of higher-order assemblies (Crosetto et al., 2008). Experimental studies demonstrated that this region contributes to the stable accumulation of WRNIP1 in nuclear foci and regulates its proper subnuclear localization during replication stress (Crosetto et al., 2008; Nomura et al., 2012; Yoshimura et al., 2022; Einig et al., 2023). Disruption of the leucine zipper region does not abolish ATPase activity but significantly reduces DDT efficiency, indicating that the C-terminus is essential for pathway coordination and substrate-specific functions rather than catalytic activity itself (Nomura et al., 2012). Loss of this region impairs the ability of WRNIP1 to accumulate properly at sites of replication stress, thereby compromising genome maintenance functions (Yoshimura et al., 2022). Together, these observations indicate that the conserved C-terminal regions of Mgs1/WRNIP1 provide structural and regulatory functions that complement the catalytic activity of the central AAA + ATPase domain during replication stress responses (Nomura et al., 2012). Whether the C-terminal region of yeast Mgs1 fulfils analogous mechanistic roles remains to be elucidated.

## 6 DNA-binding property of Mgs1 and WRNIP1

Biochemical and genetic studies indicate that Mgs1 associates with multiple DNA substrates, including stalled replication forks and non-canonical secondary DNA structures such as DNA quadruplexes (G4) (Yoshimura et al., 2009; Zacheja et al., 2020; Hegedus et al., 2024). These interactions support an emerging idea of its conserved role as a replication fork-associated factor involved in coordinating protein assemblies required for genome maintenance under replication stress conditions (Marabitti et al., 2020; Einig et al., 2023; Valenzisi et al., 2024).

DNA quadruplexes are stable non-B DNA structures that form within guanine-rich genomic regions through Hoogsteen hydrogen bonding between stacked guanine tetrads (Bochman et al., 2012). Although G4 structures can participate in physiological regulatory processes, their persistence represents a significant obstacle to replication fork progression. Experimental studies demonstrated that replicative polymerases, including Pol δ, are unable to efficiently replicate through G4-forming templates, leading to polymerase stalling and replication fork slowing (Lerner and Sale, 2019). Consequently, the unresolved G4 structures create an important endogenous source of replication stress and genome instability (Lipps and Rhodes, 2009). Among these factors, Mgs1 has emerged as a potential G4-binding protein. Biochemical studies demonstrated that Mgs1 directly associates with G4 substrates *in vitro* and preferentially localizes to genomic regions with high G4-forming potential *in vivo*, suggesting a role in the recognition or processing of these structures during DNA replication and genome maintenance. Functionally, Mgs1 contributes to genome stability by regulating G4 dynamics and preventing replication-associated stress (Zacheja et al., 2020).

Efficient resolution of G4 structures, therefore, requires the coordinated activity of specialized helicases and G4-associated regulatory proteins (Paeschke and Burkovics, 2021). Pif1 is the major G4-resolving enzyme in yeast, which actively unwind G4 structures and facilitate replication fork progression through guanine-rich regions (Paeschke et al., 2011; 2013). The coordinated action of replicative polymerase, G4-binding proteins, and helicases ensures proper replication of these structures. Although the precise molecular mechanism remains incompletely understood, current evidence points to the possibility that Mgs1 may contribute to the recognition of G4 structures, and the recruitment of G4-processing factors at the replication forks. Coordinated action of Mgs1 and Pif1 may therefore represent an important mechanism for preventing replication-associated fork stalling and genome instability imposed by persistent G4 structures (Paeschke and Burkovics, 2021). Pif1 functions not only in the nucleus but also plays an important role in the maintenance of the mitochondrial DNA. The helicase activity of Pif1 is required for efficient mitochondrial DNA replication and is indispensable for preserving mitochondrial genome integrity (Cheng et al., 2007; 2009; Muellner and Schmidt, 2020). Given the functional interaction between Pif1 and Mgs1/WRNIP1 in the nuclear genome maintenance, it is tempting to speculate that Mgs1/WRNIP1 may also participate in maintaining mitochondrial genome integrity. However, whether Mgs1/WRNIP1 has a role in the mitochondrial DNA maintenance remains an open question.

WRNIP1 binds to a replication fork mimicking DNA substrate in an ATP dependent manner and thereby hinders RAD18 binding to the DNA substrate (Yoshimura et al., 2009), which could be a potential regulatory function of WRNIP1 at the stalled replication fork. Additionally, WRNIP1 binds specifically G4 structured DNA in an ATP independent manner. WRNIP1 is indispensable for G4 resolution at the replication fork and functionally interacts with Pif1 helicase on G4 processing in humans (Hegedus et al., 2024), indicating the evolutionary conservation and importance of the G4 processing function of these proteins.

## 7 Rescue mechanisms at the stalled replication fork

Protection of stalled replication forks is essential for maintaining genome stability during replication stress. Replication fork stalling exposes regions of single-stranded DNA and generates unstable replication intermediates that are targets for nucleolytic degradation. In the absence of proper fork protection mechanisms, stalled forks may undergo resection, collapse, or aberrant recombination, which could in turn lead to chromosome instability and double-strand break formation (Liao et al., 2018; Rickman and Smogorzewska, 2019). Therefore, cells rely on coordinated fork stabilization and restart pathways to preserve fork integrity during replication stress.

DDT pathways enable ongoing DNA replication in the presence of template lesions without immediate removal of underlying DNA damage (Goodman and Woodgate, 2013; Lehner and Jinks-Robertson, 2014; Ashour and Mosammaparast, 2021). At stalled forks, pathway choice is largely coordinated by post-translational modification of PCNA (Boehm et al., 2016). Monoubiquitination of PCNA at lysine 164 by the RAD6-RAD18 ubiquitin ligase complex promotes recruitment of translesion synthesis (TLS) polymerases and facilitates direct lesion bypass (Hoege et al., 2002; Kanao and Masutani, 2017). Replicative polymerases such as DNA polymerase ε and DNA polymerase δ are unable to efficiently synthesize across many DNA lesions due to the structural constraints of their active sites (Prakash et al., 2005). TLS therefore provides an alternative damage bypass mechanism by recruiting specialised low-fidelity polymerases that can accommodate distorted DNA templates (Prakash et al., 2005; Yang and Gao, 2018). In mammalian cells, these low-fidelity polymerases are the Y-family polymerases such as Pol η, Pol ι, Pol κ, and REV1, and other low-fidelity polymerases from other families as Pol θ, Pol ζ, Pol μ, and Pol λ (Rajpurohit et al., 2025). In contrast, the major TLS polymerases in yeast are Pol η, REV1, and Pol ζ. Recruitment of Y-family polymerases to stalled replication forks is mediated through interactions with monoubiquitinated PCNA (Prakash et al., 2005; Haracska et al., 2006). Several TLS polymerases, including Pol η, Pol κ, and Pol ι, also contain PCNA-interacting peptide (PIP) domains that further stabilize their association with the replisome (Arianna and Korzhnev, 2024). In case of Pol η, recruitment to stalled forks is also facilitated by interactions with RAD18, thereby promoting efficient lesion bypass during replication stress (Yuasa et al., 2006).

In contrast to PCNA monoubiquitination, K63-linked polyubiquitination of PCNA promotes error-free DDT pathways associated with template switching (TS), which is the dominant form of DDT pathway under normal circumstances. In yeast PCNA polyubiquitilation is catalysed by Ubc13-Mms2 complex together with E3 ligases such as Rad5, whereas in mammalian cells, the functional homologues HLTF and SHPRH facilitate a similar process. Template switching enables bypass of replication-blocking lesions through utilization of the newly synthesized sister chromatid as an undamaged template, thereby providing a relatively error-free bypass mechanism (Unk et al., 2010; Kanao and Masutani, 2017).

In addition to ubiquitination, PCNA is also regulated by SUMOylation, which suppresses inappropriate recombination events during DNA replication. In yeast cells, SUMOylated PCNA recruits anti-recombinogenic helicase Srs2, thereby preventing unscheduled homologous recombination at replication forks (Hoege et al., 2002; Papouli et al., 2005; Burkovics et al., 2013). In mammalian cells, SUMOylated PCNA similarly promotes recruitment of anti-recombinogenic factors such as PARI, which inhibits RAD51-dependent recombination intermediates (Gali et al., 2012; Moldovan et al., 2012; Burkovics et al., 2016). Through these post-translational modifications, PCNA functions as a central regulatory platform that coordinates pathway choice between error-prone translesion synthesis, error-free template switching, or recombination-associated rescue pathways.

### 7.1 The function of Mgs1 at stalled replication forks

**FIGURE 2:**
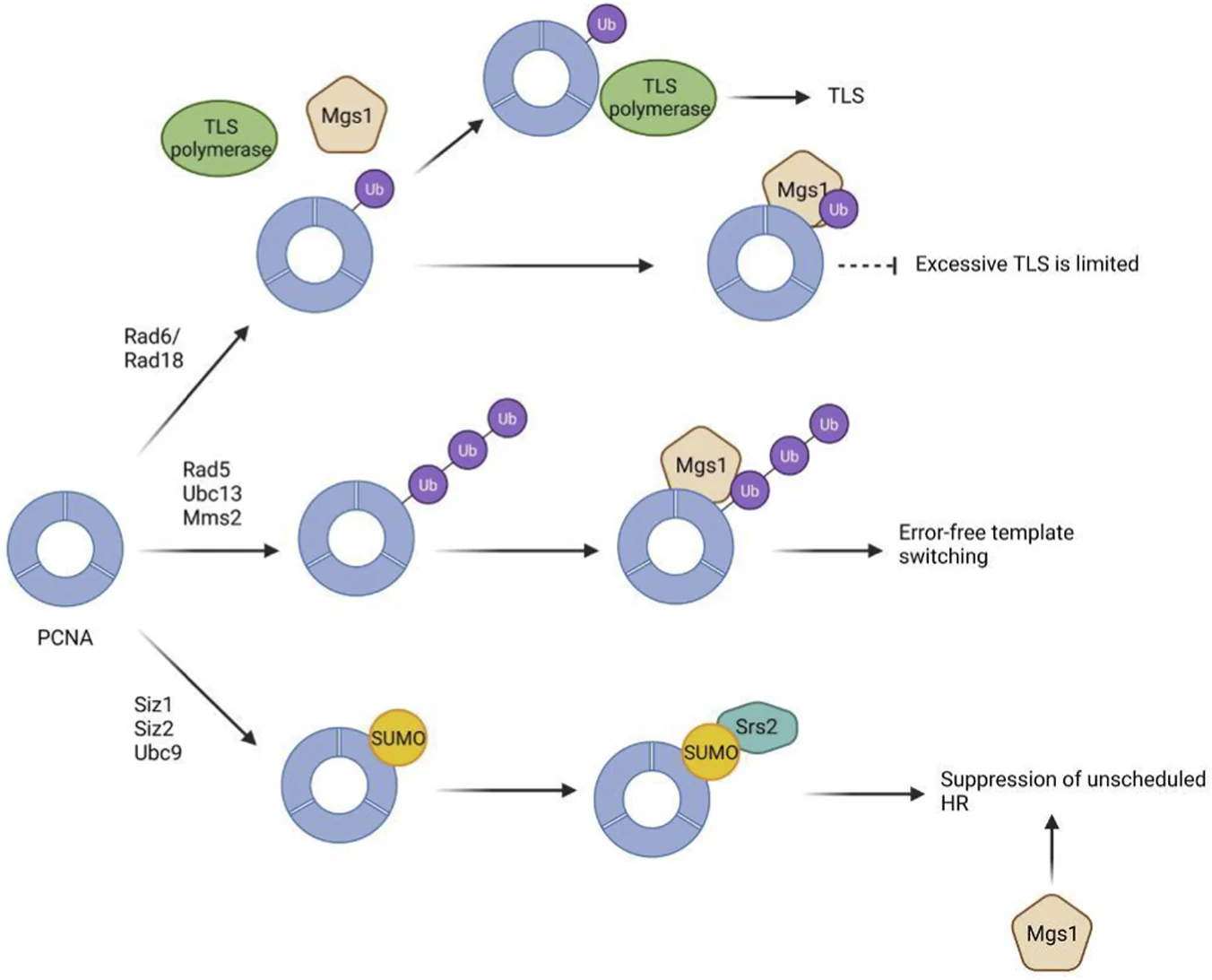
The function of Mgs1 at the stalled replication fork. Mgs1 plays multiple roles in pathway selection during replication fork rescue. Unregulated binding to monoUb-PCNA could result in excessive TLS. The binding of polyUB-PCNA by Mgs1 could direct the fork rescue into the template switching mechanism. In addition, Mgs1 suppresses inappropriate homologous recombination events, thereby contributing to genome stability.

Surprisingly not MGS1 deletion, but its overexpression sensitises yeast cells to DNA-damaging agents such as MMS, HU, and UV, indicating its contribution in replication stress response (Hishida et al., 2001). At stalled replication forks in yeast, Mgs1 is linked to the replication machinery through interactions with PCNA, particularly ubiquitylated PCNA, and with DNA polymerase δ (Branzei et al., 2002a; Hishida et al., 2006; Vijeh Motlagh et al., 2006; Saugar et al., 2012). Through these interactions, Mgs1 is thought to modulate polymerase activity, replication fork recovery, and the choice of damage-bypass pathway (Branzei et al., 2002a; Jiménez-Martín et al., 2020). Physical interactions with ubiquitylated PCNA and the modulatory effect of Mgs1 on Pol δ function in ubiquitin-dependent DNA damage bypass implicate Mgs1 as a downstream effector of the RAD6 pathway, providing a functional link between a ubiquitylated target and an effector that recognises the target in its modified form (Saugar et al., 2012) (Figure 2).

Additionally, Mgs1 has other important function in the rescue of the replication fork, since *mgs1* deletion is synthetic lethal with *rad6* deletion (Hishida et al., 2002). This finding demonstrates that, in the absence of the Rad18-dependent DDT pathway, the contribution of Mgs1 is essential for a salvage pathway to rescue the stalled replication fork. These two faces of Mgs1 in the contribution to the rescue of stalled replication fork is further supported by the fact that both deletion and overexpression of Mgs1 increase mitotic recombination frequency (Hishida et al., 2001). It has been discovered that Mgs1 is able to suppress the frequency of a HR-based salvage pathway for the rescue of the stalled replication fork (Jiménez-Martín et al., 2020) (Figure 2). However, the recombinogenic contribution of Mgs1 to fork rescue by HR is not clarified yet.

### 7.2 The function of WRNIP1 at stalled replication forks

In vertebrate cells, WRNIP1 functions at replication forks similarly to that described for yeast Mgs1, although several phenotypes identified in yeast have not yet been directly examined in vertebrate systems. Increasing evidence indicates that WRNIP1 is recruited to stalled replication forks and replication stress-associated chromatin regions, including sites of replication-transcription conflicts and R-loop accumulation (Marabitti et al., 2020; Einig et al., 2023; Valenzisi et al., 2024). WRNIP1 has also been implicated in the maintenance of genome stability at DNA secondary structures, further supporting its role as a replication stress response factor (Hegedus et al., 2024).

WRNIP1 was named after its interacting partner the Werner syndrome helicase (WRN) (Yi et al., 2001). It is known that WRN contributes to replication fork protection through multiple mechanisms (Mukherjee et al., 2018). Therefore, it is plausible that WRNIP1 also contributes to replication fork protection through similar mechanisms.

**FIGURE 3:**
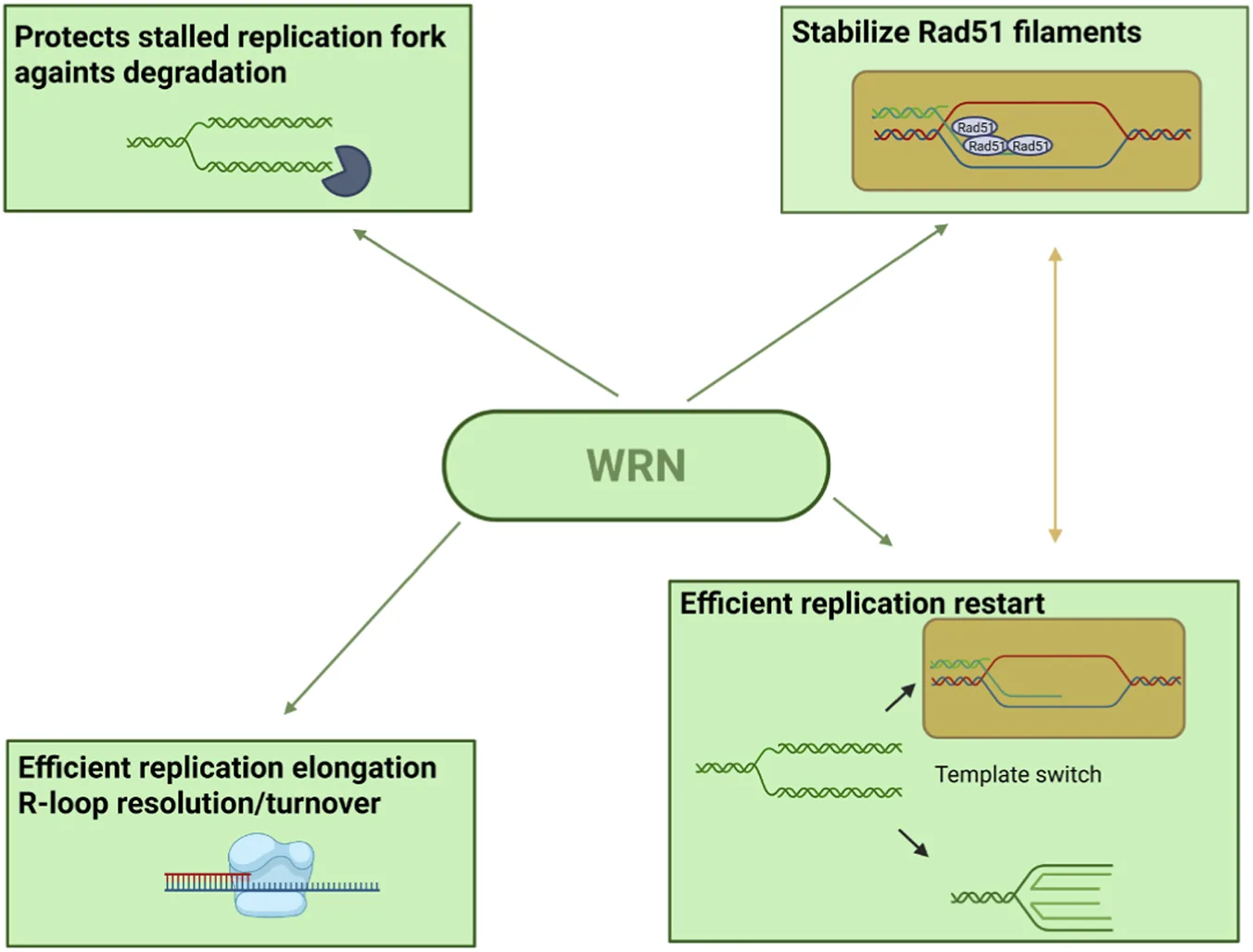
Functions of the Werner syndrome helicase at stalled replication fork. WRN participates in multiple pathways that promote replication fork stability and restart. Current evidence suggests that WRNIP1 contributes to several of the same replication fork rescue mechanisms as WRN. However, whether WRN and WRNIP1 cooperate directly in these processes has not yet been experimentally demonstrated.

WRN plays an important role in maintaining efficient replication fork progression. In the absence of WRN, replication forks become asymmetric (Rodríguez-López et al., 2002). WRN also prevents fork stalling or collapse and promotes efficient replication restart following replication stress (Sidorova et al., 2008; 2013; Ammazzalorso et al., 2010). In addition, WRN is required for efficient replication elongation, most likely *via* its G4 unwinding activity and proofreading function (Kamath-Loeb et al., 2012; Ketkar et al., 2017; Yoon et al., 2024). Furthermore, WRN suppresses double-strand break formation at stalled replication forks (Pichierri et al., 2012). Beyond its catalytic activities, WRN also stabilizes newly synthetised DNA strand through a non-catalytic DNA-binding mechanism, thereby protecting stalled replication forks against MRE11 degradation (Iannascoli et al., 2015). Moreover, similar to the WRNIP1, WRN promotes stabilization of RAD51 filaments at stalled replication forks (Iannascoli et al., 2015) (Figure 3).

Functional studies demonstrated that the integrity of key WRNIP1 domains is required for its proper recruitment and function at stressed replication forks. Deletion or mutation of the UBZ domain impairs WRNIP1 accumulation at sites of replication stress and disrupts its interaction with ubiquitinated PCNA, which could mean that ubiquitin-dependent recruitment is important for WRNIP1 localization during replication stress responses (Bish and Myers, 2007; Crosetto et al., 2008; Nomura et al., 2012; Socha et al., 2020; Valenzisi et al., 2024). Similarly, disruption of the C-terminal leucine zipper region abolishes WRNIP1 nuclear foci formation and reduces cellular resistance to replication-associated DNA damage without affecting the ATPase activity, suggesting that oligomerization-dependent protein interactions are required for efficient fork protection function (Crosetto et al., 2008; Nomura et al., 2012; Yoshimura et al., 2022; Einig et al., 2023). WRNIP1 has important function in the protecting stalled replication fork against MRE11, DNA2, or SLX4 dependent replication fork degradation (Leuzzi et al., 2016; Porebski et al., 2019; Shi et al., 2023).

In vertebrate cells, WRNIP1 participates in RAD18-associated replication stress response pathways, although its functions appear broader than canonical DNA damage tolerance alone. Early biochemical studies demonstrated physical and functional interactions between WRNIP1 and RAD18 at replication fork-like DNA substrates (Yoshimura et al., 2009), suggesting coordinated regulation of ubiquitin-dependent replication stress responses. WRNIP1 associates with forked DNA structures and modulates RAD18 interactions with stalled fork intermediates, supporting a functional relationship between these factors during replication-associated DNA damage processing (Yoshimura et al., 2009). Additional studies demonstrated the role of WRNIP1 beyond canonical RAD18-dependent translesion synthesis pathways. Under replication stress, WRNIP1 was shown to cooperate with RAD18 and ATMIN in promoting ATM signalling at stalled replication forks (Kanu et al., 2016). Depletion of WRNIP1 impairs replication stress-induced ATM activation and increases chromosome instability, pointing to WRNIP1 function as an important mediator connecting ubiquitin-dependent replication stress sensing to checkpoint activation pathways (Kanu et al., 2016).

Recent studies also elucidated a role of WRNIP1 in the suppression of R-loop-associated genome instability. In human cells experiencing replication-transcription conflicts, WRNIP1 was shown to stabilize RAD51 at sites of R-loop accumulation and promote ATM-dependent signalling in response to replication stress (Marabitti et al., 2020; Valenzisi et al., 2024). Loss of WRNIP1 resulted in elevated R-loop accumulation, increased DNA damage signalling, and elevated genomic instability, particularly under impaired ATR checkpoint activity conditions. Additional studies demonstrated that WRNIP1 is enriched at difficult-to-replicate genomic regions containing DNA secondary structures, including G4-prone loci, further supporting its role in protecting stressed replication forks from in proper processing (Hegedus et al., 2024).

Further strengthening WRNIP1 contribution to the damage bypass processes is its interaction with DNA polymerase η (Yoshimura et al., 2014; 2022). It has been described, that WRNIP1 acts upstream of it, most likely by inhibiting a PrimPol-dependent alternative UV-bypass pathway, therefore, in the absence of WRNIP1 the PrimPol can be activated at UV-induced lesions (Yoshimura et al., 2014). This hypothesis is supported by the observation that WRNIP1 overexpression promotes PrimPol degradation (Yoshimura et al., 2019), suggesting that WRNIP1 may regulate pathway choice by preferentially channelling UV bypass towards the Ub-PCNA and DNA polymerase η-mediated error-free pathway.

Very recently it has been demonstrated, that WRNIP1 cooperates with WRN helicase to promote the assembly of a translesion synthesis complex following UV irradiation, thereby facilitating efficient fork progression across UV-induced lesions. In addition to promoting TLS complex assembly, the exonuclease activity of WRN enhances the fidelity of Rev1- and DNA polymerase η-dependent damage bypass (Yoon et al., 2024). Moreover, the ATPase domain of WRNIP1 was shown to play a critical role in maintaining the fidelity of Y-family DNA polymerases (Yoon et al., 2025). Cells expressing ATPase-deficient WRNIP1 or WRN display increased misincorporation opposite UV-induced DNA lesions, indicating that the ATPase activities of both proteins contribute to accurate translesion DNA synthesis. Surprisingly, simultaneous disruption of ATPase activities of WRNIP1 and WRN results in an additive increase in mutation frequency, indicating that WRNIP1 performs additional functions in promoting high fidelity TLS of Y-family polymerases (Yoon et al., 2025; Yoon et al., 2025). One possible mechanistic explanation is that WRNIP1 facilitates the recruitment or stabilization of WRN helicase, consistent with earlier *in vitro* studies demonstrating direct interaction between the two proteins (Kanamori et al., 2011).

The studies mentioned above allow to suggest that WRNIP1 evolved from a primarily RAD6/RAD18-associated DDT factor into a multifunctional replication stress response regulator that coordinates ubiquitin signalling, checkpoint activation, fork stabilization, and the processing of difficult-to-replicate DNA structures.

## 8 WRNIP1 is connected to Fanconi anaemia pathway

Fanconi anaemia is a rare genetic disorder, characterized by congenital abnormalities, progressive bone marrow failure and markedly increased predisposition to cancer. Fanconi anaemia pathway, constituted by Fanconi anaemia complementation group members and Fanconi anaemia associated proteins, is the principal mechanism responsible for the interstrand crosslinked DNA (ICL) repair. Fanconi anaemia core complex recognizes the interstrand crosslink and promotes the monoubiquitylation of FANCI/FANCD2 complex (Nalepa and Clapp, 2018; Niraj et al., 2019; Lemonidis et al., 2022). This bi-monoubiquitylation promotes stable association of the ID complex with DNA, allowing it to function as a sliding clamp that coordinates the recruitment of nucleases, translesion synthesis polymerases, homologous recombination factors, and other downstream repair proteins required for ICL resolution (Niraj et al., 2019; Wang et al., 2020).

More recently, WRNIP1 was identified as a member of Fanconi D2 complex, suggesting that it also contributes to the initiation of the interstrand crosslink repair pathway. Socha et al. demonstrated that WRNIP1 promotes efficient chromatin loading of the FANCD2/FANCI complex through a mechanism, dependent on its UBZ domain (Socha et al., 2020), suggesting ubiquitin-mediated interaction in the early stages of ICL repair. These findings suggest that WRNIP1 may facilitate the recruitment or stabilization of the ID complex at sites of ICL damage rather than directly regulating its catalytic activation. However, the precise role of WRNIP1 in the FA pathway has not yet been understood completely. Although WRNIP1 physically associates with FANCD2, recent work showed that loss of WRNIP1 or its UBZ domain do not impair FANCD2 monoubiquitination (Valenzisi et al., 2024), indicating that WRNIP1 is not required for activation of the FA core complex itself. Instead, WRNIP1 may function downstream of FANCD2 monoubiquitination by promoting efficient chromatin association, coordinating downstream repair factors, or facilitating replication fork stabilization during ICL repair. Further mechanistic studies will therefore be required to elucidate how WRNIP1 contributes to Fanconi anaemia pathway-mediated genome maintenance.

## 9 The role of WRNIP1 in transcription - replication conflict

There is growing evidence that WRNIP1 also plays an important role in the resolution of transcription - replication conflicts (TRCs) (Marabitti et al., 2020; 2022; Valenzisi et al., 2024). R-loops are DNA:RNA hybrids, that influence replication fork progression. Dysregulated R-loops turnover could lead to either co-directional or head-on collisions between the replisome and RNA polymerase, ultimately resulting in replication stress. Under normal conditions, R-loop formation activates ATR dependent checkpoint, that promotes their timely resolution (Barroso et al., 2019). In the absence of functional ATR-mediated R-loop resolution, the chromatin-bound fraction of WRNIP1 increases dramatically, suggesting that WRNIP1 could represented an alternative R-loop resolution pathway to ensure genomic integrity. Interestingly, WRNIP1 recruitment to chromatin depends on the presence of the WRN helicase, although the two proteins appear to function independently in R-loop resolution (Marabitti et al., 2020). Since WRN is not active on DNA:RNA hybrid substrates *in vitro* (Chakraborty and Grosse, 2011), it is likely that WRNIP1 cooperates with another helicase on R-loop resolution. Alternatively, WRNIP1 may bind to R-loops, thereby protecting stalled replication forks against double-strand break formation. Consistent with this hypothesis, loss of WRNIP1 results in the accumulation of transcription-dependent DNA damage. Furthermore, overexpression of RNaseH1, which degrades DNA:RNA hybrids, or inhibition of transcription, suppresses the DNA damage phenotype caused by WRNIP1 deficiency (Valenzisi et al., 2024).

Further supporting the importance of WRNIP1 in the resolution of TRCs is its interaction with RNA polymerase II (RNAPII). RNAPII serves as an important platform for the recruitment of R-loop resolving factors to sites of TCRs (Shivji et al., 2018; Einig et al., 2023). It has been demonstrated that the E3 ubiquitin ligase HUWE-1 also plays a key role in this process. HUWE-1 promotes the association between RNAPII and WRNIP1, and this interaction depends on its ubiquitin ligase activity, ultimately suppressing ATM-dependent checkpoint activation (Einig et al., 2023).

Taken together, these findings demonstrate that WRNIP1 plays an important role in the resolution of transcription-replication conflicts. However, the precise molecular mechanism by which WRNIP1 contributes to this process remains to be elucidated.

## 10 Conclusion and further perspectives

Mgs1/WRNIP1 proteins are highly conserved throughout eukaryotic evolution, highlighting the fundamental importance of this protein family in maintaining genome stability during DNA replication. Although vertebrate WRNIP1 has acquired additional interactions and functions associated with replication stress signalling, the core molecular activities of Mgs1/WRNIP1 appear to be evolutionarily conserved. In both yeast and vertebrates, Mgs1/WRNIP1 functions at stalled replication forks through interactions with proliferating cell nuclear antigen (PCNA) (Hishida et al., 2006; Saugar et al., 2012), regulation of several PCNA-associated proteins such as DNA polymerase δ (Branzei et al., 2002a; Yoshimura et al., 2026) and DNA polymerase η (Yoshimura et al., 2014), and interaction with DNA helicases (Kawabe et al., 2006; Kanamori et al., 2011; Zacheja et al., 2020). This specialized interaction network (DNA polymerases–PCNA (and its modifications) – DNA helicases) is unique, pointing to Mgs1/WRNIP1 being a potential multifunctional regulatory factor that coordinates protein assemblies at stressed replication forks, as well as fine-tunes DDT pathways.

This regulatory role can be observed for Mgs1, which suppresses an alternative recombination-dependent mechanism of replication fork rescue. When the canonical error-free DDT pathway is compromised, loss of Mgs1 permits activation of this normally restricted recombination-mediated pathway, allowing stalled replication forks to be rescued through an alternative mechanism (Jiménez-Martín et al., 2020). To date an analogous regulatory role of WRNIP1 has not been demonstrated. In budding yeast, simultaneous deletion of MGS1 and RAD6 is synthetic lethal (Hishida et al., 2002), implicating that Mgs1 becomes indispensable for efficient fork rescue and cell viability when the canonical DDT is compromised. In contrast, double deletion of WRNIP1 and RAD18 in chicken DT40 cells leads to a proliferation defect but not synthetic lethality (Yoshimura et al., 2006). This suggests that the functional relationship between Mgs1/WRNIP1 and RAD6/RAD18-associated DDT pathways has diverged during evolution. One possible explanation is that there is a greater redundancy in replication stress responses in vertebrate cells than in budding yeast. Consistent with this idea, PCNA ubiquitination is indispensable for error-free DDT in yeast (Torres-Ramos et al., 1996), whereas vertebrate cells retain additional mechanisms that support replication fork progression and restart even when PCNA ubiquitination is impaired (Krijger et al., 2011; Abe et al., 2018). Hence, loss of WRNIP1 may be compensated by other replication stress response factors, reducing the severity of the phenotype in vertebrate cells.

Despite considerable progress in understanding cellular functions of Mgs1/WRNIP1, the functional roles of the individual domains have not been defined completely. Current evidence suggests that the N-terminal UBZ domain is primarily responsible for targeting Mgs1/WRNIP1 to ubiquitinated proteins, in particular ubiquitinated PCNA, thereby facilitating the recruitment to replication stress sites (Crosetto et al., 2008). The ATPase domain, on the other hand, is important for the functions of the protein, however the mechanistic aspect of its activity is unclear. Despite its relatively weak ATPase activity (Zacheja et al., 2020), mutations impairing ATP binding or hydrolysis result in significant defects in DDT and replication fork maintenance (Hishida et al., 2001; Branzei et al., 2002b).

Similarly, little is known about the molecular interfaces of Mgs1/WRNIP1 involved in protein-protein and protein-DNA interactions. While the PCNA-interacting peptide (PIP) motif and the ubiquitin-binding properties of the UBZ domain have been characterized, the DNA-binding regions of Mgs1/WRNIP1 remain unidentified (Hishida et al., 2006; Saugar et al., 2012). Likewise, the C terminal leucin zipper is often considered to mediate protein oligomerization and protein-protein interactions, its binding partners and structural functions have not been examined systematically yet. Addressing these questions through structural and biochemical studies will be essential for understanding how Mgs1/WRNIP1 coordinates protein assemblies that regulate replication fork stability.

**FIGURE 4:**
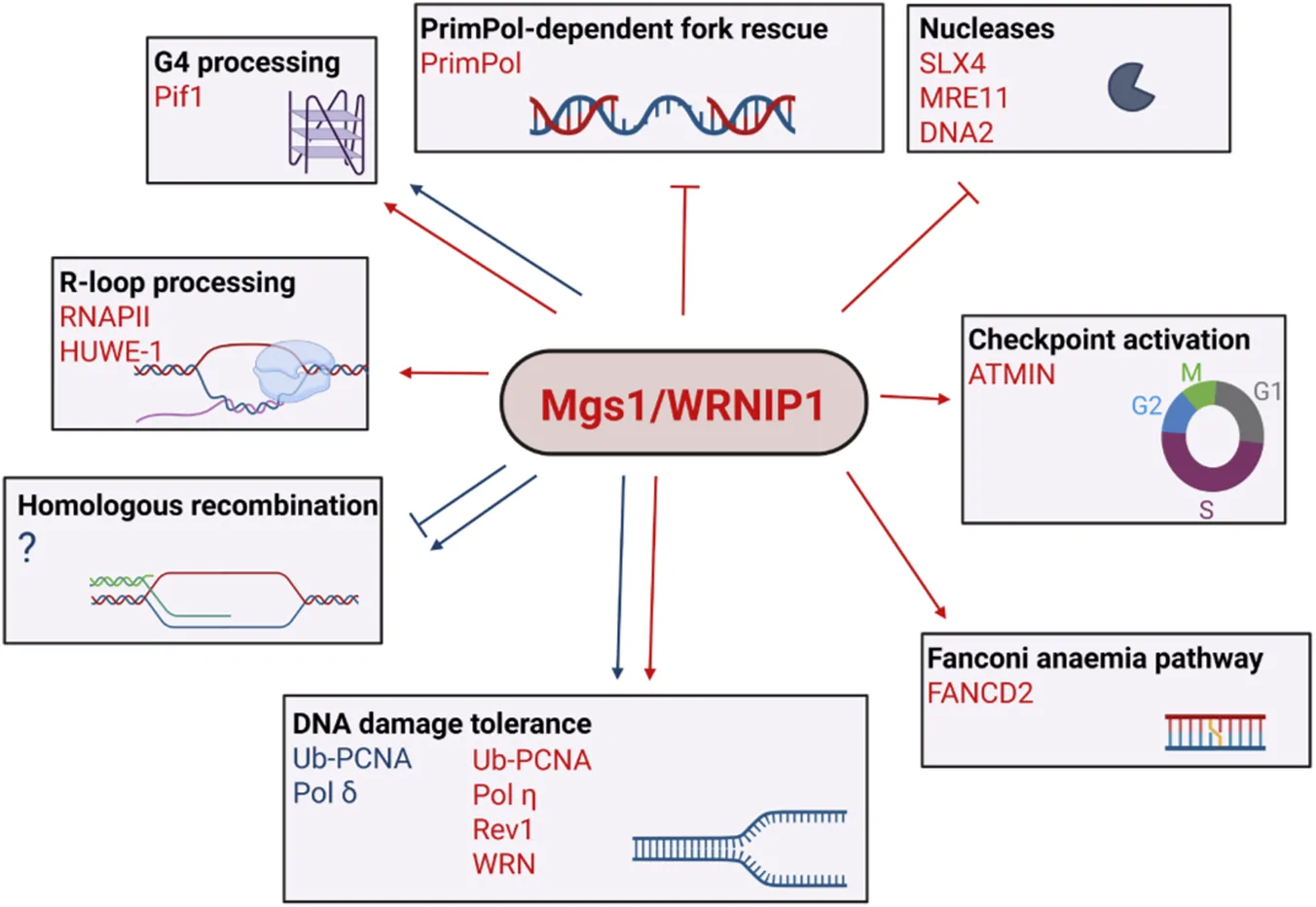
The functions of Mgs1/WRNIP1 are coupled with several different mechanisms at the stalled replication fork. The central position of Mgs1/WRNIP1 at the stalled replication fork determines its regulatory or fine-tuning function, which can contribute to the selection between the different actions at stalled replication forks.

The available evidence suggests that Mgs1/WRNIP1 may function as a central regulator of DNA repair pathway choice during replication stress (Figure 4). Several observations support this model. First, Mgs1/WRNIP1 is recruited to PCNA, the principal regulatory platform that coordinates DNA replication and DNA damage tolerance at stalled replication forks. Second, through its UBZ domain, Mgs1/WRNIP1 recognizes ubiquitinated forms of PCNA, particularly polyubiquitinated PCNA, which acts as a main molecular signal directing pathway choice within the DNA damage tolerance network. Third, Mgs1/WRNIP1 interacts with numerous proteins involved in distinct genome maintenance pathways, including the FANCD2–FANCI complex of the Fanconi anaemia pathway, the homologous recombination factors RAD51 and BRCA2, the SLX1–SLX4 structure-specific nuclease complex, the DNA helicases PIF1 and WRN, and the ubiquitin ligases RNF166 and HUWE1, as well as the replication stress signalling factor ATMIN.

We propose that Mgs1/WRNIP1 functions as a molecular coordinator of replication stress responses rather than as a dedicated repair factor. By integrating ubiquitin-dependent signalling with interactions involving DNA polymerases, helicases, and DNA repair complexes, Mgs1/WRNIP1 fits well to influence pathway choice at stalled replication forks. Defining the molecular mechanisms that regulate these interactions will be crucial for understanding how cells maintain genome stability while balancing the efficiency and fidelity of DNA damage tolerance.
